# Apoptosis of Endothelial Cells by 13-HPODE Contributes to Impairment of Endothelial Barrier Integrity

**DOI:** 10.1155/2016/9867138

**Published:** 2016-10-12

**Authors:** Valerie E. Ryman, Nandakumar Packiriswamy, Lorraine M. Sordillo

**Affiliations:** College of Veterinary Medicine, Michigan State University, East Lansing, MI 48824, USA

## Abstract

Inflammation is an essential host response during bacterial infections such as bovine mastitis. Endothelial cells are critical for an appropriate inflammatory response and loss of vascular barrier integrity is implicated in the pathogenesis of* Streptococcus uberis*-induced mastitis. Previous studies suggested that accumulation of linoleic acid (LA) oxygenation products derived from 15-lipoxygenase-1 (15-LOX-1) metabolism could regulate vascular functions. The initial LA derivative from the 15-LOX-1 pathway, 13-hydroperoxyoctadecadienoic acid (HPODE), can induce endothelial death, whereas the reduced hydroxyl product, 13-hydroxyoctadecadienoic acid (HODE), is abundantly produced during vascular activation. However, the relative contribution of specific LA-derived metabolites on impairment of mammary endothelial integrity is unknown. Our hypothesis was that* S. uberis*-induced LA-derived 15-LOX-1 oxygenation products impair mammary endothelial barrier integrity by apoptosis. Exposure of bovine mammary endothelial cells (BMEC) to* S. uberis* did not increase 15-LOX-1 LA metabolism. However,* S. uberis* challenge of bovine monocytes demonstrated that monocytes may be a significant source of both 13-HPODE and 13-HODE during mastitis. Exposure of BMEC to 13-HPODE, but not 13-HODE, significantly reduced endothelial barrier integrity and increased apoptosis. Changing oxidant status by coexposure to an antioxidant during 13-HPODE treatment prevented adverse effects of 13-HPODE, including amelioration of apoptosis. A better understanding of how the oxidant status of the vascular microenvironment impacts endothelial barrier properties could lead to more efficacious treatments for* S. uberis* mastitis.

## 1. Introduction

Inflammation contributes to a variety of human and veterinary diseases, including mastitis. Bovine mastitis caused by* Streptococcus uberis* results in severe damage to milk-producing tissues as a result of an uncontrolled inflammatory response. Previous clinical and histopathological data suggested that disruption of the endothelial barrier contributed to disease pathology. For example,* S. uberis* intramammary challenge studies reported a loss in the blood-milk barrier as indicated by a sustained increase of plasma proteins in milk [1]. Similarly, histopathological analysis demonstrated that neutrophils accumulated in mammary tissue up to several days after* S. uberis* intramammary challenge indicating an inability of the endothelium to limit leukocyte influx across the blood-milk barrier [2, 3]. Subcutaneous edema after intramammary* S. uberis* challenge also indicated an inability to preserve a selectively permeable vascular barrier [4]. The mechanisms that may cause endothelial dysfunction during* S. uberis* mastitis are undefined, but some fatty acid-derived oxylipids were implicated in contributing to development of dysfunctional endothelial responses [5, 6].

Oxylipids are synthesized from esterified polyunsaturated fatty acids (PUFA) that are cleaved from the phospholipid membrane by cytosolic phospholipase A_2_. Cleaved PUFA are often oxidized through several different enzymatic pathways including 15-lipoxygenase (15-LOX) [7, 8]. Initial enzymatic oxygenation products can be further enzymatically metabolized by a variety of downstream enzymes including hydrolases and dehydrogenases [9, 10]. Additionally, initial oxygenation products during oxylipid biosynthesis may be reduced depending on the redox status of the cellular environment. For example, enzymatic oxidation of linoleic acid (LA) by 15-LOX-1 predominately yields 13-hydroperoxyoctadecadienoic acid (13-HPODE) and can be reduced to 13-hydroxyoctadecadienoic acid (13-HODE) by antioxidants and reducing agents, such as glutathione [11]. Dehydrogenation of 13-HODE to an anti-inflammatory 13-oxooctadecadienoic acid (13-oxoODE) can occur by the action of NADPH-dependent fatty acid dehydrogenases [9]. Since LA oxidation and metabolism is a sequential process, 13-HPODE biosynthesis is required for the subsequent generation of 13-HODE and 13-oxoODE. Current literature supports an important role for some LA-derived 15-LOX-1 oxylipids in a normal inflammatory response [12–14]. However, previous data also suggested 13-HPODE biosynthesis induced death of various cell types and was associated with the pathology of severe inflammatory based diseases [15, 16].

A recently published study by the current authors proposed that LA-derived oxylipids might be responsible for contributing to* S. uberis* pathogenesis [17]. Authors specifically highlighted the potential contribution of the 15-LOX-1 oxidation pathway to disruption of the endothelial barrier [17]. Various studies showed that 13-HODE induced vascular activation and could play a role in regulating endothelial barrier integrity during inflammation [6, 18]. The initial 15-LOX-1 LA oxygenation product, 13-HPODE, was implicated in contributing to apoptosis of endothelial cells, which could be a key event contributing to endothelial dysfunction [19–21]. However, the capacity of specific LA oxygenation products to compromise the mammary endothelial barrier during* S. uberis* mastitis is unknown. Thus, the hypothesis for the current study was that* S. uberis*-induced LA-derived 15-LOX-1 oxygenation products impair mammary endothelial barrier integrity by apoptosis.

## 2. Materials and Methods

### 2.1. Reagents

High performance liquid chromatography- (HPLC-) grade acetonitrile, HPLC-grade methanol, formic acid, sodium selenite, insulin, heparin, transferrin, ethylenediaminetetraacetic acid (EDTA), triphenylphosphine (TPP), sodium selenite, soybean lipoxidase type V, and linoleic acid were purchased from Sigma–Aldrich (St. Louis, MO). Diethyl ether and butylated hydroxy toluene (BHT) were purchased from ACROS Organics (Fair Lawn, NJ). YOPRO-1 and propidium iodide stains were from Thermo Fisher Scientific (Waltham, MA). Antibiotics/antimycotics, trypsin-EDTA, glutamine, and bovine collagen were from Life Technologies (Carlsbad, CA). All predesigned bovine TaqMan® primers were purchased from Applied Biosystems (Foster City, CA). Deuterated oxylipid standards, nondeuterated oxylipid standards, and indomethacin were purchased from Cayman Chemical (Ann Arbor, MI). Magnesium sulfate was purchased from Avantor Performance Materials, Inc. (Central Valley, PA), and sodium borate from Fisher Science Education (Nazareth, PA). Fetal bovine serum was purchased from Hyclone Laboratories, Inc. (Logan, Utah). The HEPES buffer, HAM's F-12k, and RPMI 1640 were from Corning Inc. (Corning, NY).

### 2.2. Preparation of* Streptococcus uberis* for* In Vitro* Challenge

*Streptococcus uberis* was streaked onto a blood agar plate to enable collection of 3 pure colony forming units (CFU). The 3 CFU were added to 100 mL RPMI 1640 medium containing 5% fetal bovine serum (FBS), 300 mg/mL L-glutamine, and 0.1 *μ*M sodium selenite. The suspension was shaken at 37°C for 13 hr. For BMEC challenge experiments, the bacterial suspension was centrifuged at 11,000 ×g for 30 min at 4°C. After centrifuging, the supernatant was filtered through a filter with a pore size of 0.22 *μ*M. For monocyte challenge experiments, filtered supernatant was utilized as described and also bacterial suspension was heat-killed in a 65°C water bath for 45 min. Efficacy of heat-killing was determined by restreaking neat bacterial suspension onto a blood agar plate and incubating at 37°C to confirm absence of* S. uberis* growth.

### 2.3. Primary Bovine Cell Isolation and Culture

#### 2.3.1. Isolation and Culture of Primary Bovine Mammary Endothelial Cells (BMEC)

Mammary endothelial cells were collected from the supramammary artery of healthy Holstein dairy cows based on techniques described previously [22]. The BMEC were purified by limited cloning techniques and then cultured in BMEC media containing Ham's F-12K medium containing 10% FBS, 20 mM HEPES, antibiotics and antimycotics (100 U/mL consisting of penicillin, streptomycin, and amphotericin B), heparin (100 *μ*g/mL), insulin (10 *μ*g/mL), transferrin (5 *μ*g/mL), and sodium selenite (10 ng/mL). Cells were revived from liquid nitrogen at pass 4 and used up to passage 10. For experiments involving exposure to* S. uberis*, cells were seeded the day before at 2.5 × 10^6^ cells/100 mm cell culture dish. The* S. uberis* supernatant was diluted 1 : 3 in BMEC media for challenge. At time of challenge, all media were aspirated from 100 mm dishes and replaced with diluted* S. uberis* supernatant. The BMEC then were incubated for 4 hr and 36 hr at 37°C. Lipopolysaccharide (LPS) at 25 ng/mL was added for 4 hr as a positive control.

#### 2.3.2. Isolation and Culture of Primary Bovine Monocytes

Peripheral blood mononuclear cells (PBMC) were collected from healthy Holstein dairy cows by methods previously described [23]. Primary monocytes were isolated from PBMC by the plate adherence method in RPMI 1640 medium containing 5% fetal bovine serum (FBS), 300 mg/mL L-glutamine, antibiotics and antimycotics (100 U/mL consisting of penicillin, streptomycin, and amphotericin B), and 0.1 *μ*M sodium selenite [24]. The PBMC and monocytes were collected and prepared fresh for each replicate. For* S. uberis* challenge of monocytes, PBMC were seeded at a concentration of 8 × 10^7^ cells/100 mm cell culture dish with approximately 10% cell adherence after 3 hr incubation at 37°C and 3 subsequent washes. Percentage of monocytes from adherent cell population was assessed by flow cytometry based on previous work in our lab [25] and in the current study was approximately 75% monocytes. After the washes, either* S. uberis* supernatant or heat-killed* S. uberis* was added for 4 hr and 36 hr. The multiplicity of infection for heat-killed* S. uberis* challenge was an average of 54 : 1. The* S. uberis* supernatant was diluted 1 : 3 for challenge. Monocytes were incubated for 4 hr and 36 hr at 37°C. Lipopolysaccharide at 25 ng/mL was added for 4 hr as a positive control.

### 2.4. Primary BMEC and Monocyte mRNA Quantification

Total mRNA was isolated with the RNeasy Mini Kit (Qiagen, Venlo, Limburg) following the manufacturer's instructions. The qRT-PCR was completed using predesigned TaqMan minor groove binding primers with FAM probe from Applied Biosystems (Table 1). Reaction mixtures included TaqMan Gene Expression Master Mix, cDNA, and TaqMan Gene Expression Assay Mix that contained primer for gene of interest. Samples were run in triplicate with *β*-actin, TATA-box binding protein (TBP), and phosphoglycerate kinase 1 (PGK1) as endogenous controls. Target genes to assess BMEC inflammatory phenotype were cyclooxygenase-2 (COX-2), 15-LOX-1, vascular cell adhesion molecule-1, intercellular adhesion molecule-1, and interleukin-8 (IL-8). Thermal cycling conditions for BMEC were as follows: stage 1, 95°C for 20 s; stage 2, 95°C for 3 s; stage 3, 60°C for 30 s, with 40 replications through stages 2 and 3. Primary monocyte cDNA required amplification and was amplified using TaqMan PreAmp Kit (Applied Biosystems Inc.). Target genes to assess monocyte inflammatory phenotype were COX-2, 15-LOX-1, inducible nitric oxide synthase (iNOS), IL-10, and IL-6. Thermal cycling conditions for monocytes were as follows: stage 1: 50°C for 2 min, stage 2: 95°C for 10 min, stage 3: 95°C for 15 s, and stage 4: 60°C for 1 min, with 40 replicates of stages 3 and 4. Gene expression was calculated using ΔCt method for statistical analysis and also using 2^−ΔΔCt^ method for graphical purposes [26, 27].

### 2.5. Extraction and Quantification of Oxylipids

Oxylipids were extracted from the supernatant after* S. uberis* challenge in monocytes and BMEC based on techniques previously described [28]. Briefly, supernatant was collected and an antioxidant reducing agent at 4 *μ*L/mL and a mixture of internal standards containing 0.01% formic acid was added. Antioxidant and reducing agent was prepared with 50% MeOH, 25% EtOH, and 25% HPLC-grade water containing 0.54 mM EDTA 0.9 mM BHT, 3.2 mM TPP, and 5.6 mM indomethacin. The internal standards mixture contained the following deuterated oxylipids (0.1 ng/*μ*L, 10 ng total): LTB_4-*d*4_, TxB_2-*d*4_, PGF_2*α*-*d*4_, PGE_2-*d*4_,PGD_2-*d*4_, 13(S)-HODE_-*d*4_, 6-keto PGF_1*α*-*d*4_, 9(S)-HODE_-*d*4_, 12(S)-HETE_-*d*8_, and 15(S)-HETE_-*d*8_. The solution was brought up to 60% (v/v) methanol to facilitate protein precipitation. Samples were centrifuged at 4000 ×g for 30 min at 4°C. Supernatant was aspirated and diluted to 5% (v/v) methanol and kept cold prior to extraction through column cartridges prior to passing samples. the Phenomenex Strata-X 33u Polymeric Reverse-Phase Columns (500 mg/12 mL, Phenomenex, Torrance, CA) were conditioned with 6 mL methanol then 6 mL water. After conditioning, samples were run through the column and then washed with 40% methanol. The column was dried completely and then oxylipids were eluted from the columns in methanol/acetonitrile (50 : 50; v/v). Samples were dried in a Savant SVD121P SpeedVac (Thermo Scientific, Waltham, MA), resuspended in acetonitrile/water/formic acid (37 : 63 : 0.02; v/v/v), and centrifuged at 14,000 ×g for 30 min. Supernatant was aspirated and transferred to chromatography vials prior to liquid chromatography-mass spectrometry (LC-MS) quantification. Oxylipids were quantified according to previously described methods [17].

### 2.6. Preparation of 13-HPODE

The 13-HPODE was prepared according to previous reports with modifications on a Shimadzu LC-photo diode array detector system (Kyoto, Japan) [29]. Briefly, 30 mL 0.15 M (pH 9) sodium borate buffer was mixed with 50 mg of LA and 300,000 U of soybean lipoxidase type V. The suspension was stirred for 1 h on ice. The oxidation reaction was stopped by lowering the pH to 3 with 1N HCl. Immediately, 60 mL HPLC diethyl ether was added and extracts were washed with 30 mL water. After separation and dispensing of water, the extracts were dried over magnesium sulfate to remove remaining water. Extracts were resuspended in 2 mL of HPLC-grade hexane : isopropanol (96.1 : 3.9 v/v) and injected in 200 *μ*L aliquots onto a 250 × 4.6 mm Luna column (Phenomenex, Torrance, CA) at room temperature. The prepared 13-HPODE fraction was collected based on preinjected 13-HPODE standard. The method used an isocratic mobile phase (hexane : isopropanol, 96.1 : 3.9 v/v) with a flow rate of 6 mL/min. A linear 13-HPODE standard curve (0.48–300 *μ*M) was generated on a reverse-phase LC on a Waters Acquity UPLC BEH C18 1.7 *μ*M column (2.1 × 100 mm). The flow rate was 0.6 mL/min at 35°C and the quadrupole MS was in electrospray negative ionization mode. The voltage was −3 kV with the turbo ion spray source temperature at 450°C. The mobile phase was acetonitrile : MeOH : water : formic acid (47.4 : 15.8 : 26.8 : 0.01 v/v/v/v) and had an analysis time of 10 min. The concentration of 13-HPODE in the synthesized and collected fraction was quantified by Waters Empower Z software (Waters, Milford, MA) according to the 5-point standard curve.

### 2.7. Electric Cell-Substrate Impedance Sensing Assay: Endothelial Barrier Integrity

For assessment of endothelial barrier integrity, BMEC were plated on bovine collagen-coated wells with gold electrodes and grown to confluence. Electric currents passing through the monolayer were continuously measured by the Electric Cell-Substrate Impedance Sensing system (ECIS, Applied Biophysics, Inc., Troy, NY). Approximately 4–6 hr prior to treatment addition, media were changed to 0% FBS Ham's F12k media containing 20 mM HEPES, antibiotics and antimycotics (100 U/mL consisting of penicillin, streptomycin, and amphotericin B), heparin (100 *μ*g/mL), insulin (10 *μ*g/mL), transferrin (5 *μ*g/mL), and sodium selenite (10 ng/mL). Resistance across the monolayer was monitored up to 24 hr after treatment addition. Resistance was normalized to the time point immediately prior to treatment addition.

### 2.8. Measurement of Apoptosis and Necrosis

Apoptosis and necrosis of BMEC exposed to 13-HPODE or H_2_O_2_ were measured using costaining with YOPRO-1 and propidium iodide from a commercial kit (Thermo Fisher Scientific, Waltham, MA). Briefly, BMEC were seeded in 100 mm cell culture dishes overnight. The media were then changed to 0% FBS media containing 20 mM HEPES, antibiotics and antimycotics (100 U/mL consisting of penicillin, streptomycin, and amphotericin B), heparin (100 *μ*g/mL), insulin (10 *μ*g/mL), transferrin (5 *μ*g/mL), and sodium selenite (10 ng/mL) for approximately 4 hr. Treatments were added for 6 hr and 24 hr depending on experiment. Fluorescence was determined by flow cytometry according to manufacturer's protocols. Amount of apoptosis or necrosis was expressed as fold change over media control. Apoptosis was also measured by the Apo-ONE® Homogeneous Caspase-3/7 kit (Promega, Madison, WI) according to manufacturer's protocols at 6 hr after treatment. Amount of apoptosis or necrosis was expressed as fold change over media control.

### 2.9. Statistical Analysis

Differences in BMEC mRNA expression of select oxylipid biosynthetic enzymes, select adhesion molecules, and a chemotactic cytokine between respective time point controls and treatments (LPS and* S. uberis* supernatant) were determined by Student's* t*-tests. Differences in oxylipid biosynthesis for BMEC between control and treatments at each time point were tested in the same manner. Similarly, differences in bovine monocyte mRNA expression of select oxylipid biosynthetic enzymes, a marker of monocyte activation, and inflammatory cytokines between respective time point controls and LPS treatments were determined by Student's* t*-tests. Differences between control,* S. uberis* supernatant, and heat-killed* S. uberis* among time points were determined by ordinary one-way ANOVA with Tukey's* post hoc* correction. Differences in oxylipid biosynthesis for bovine monocytes exposed to* S. uberis* supernatant and heat-killed* S. uberis* were tested in the same manner. To determine differences between treatment groups (e.g., control and 13-HPODE) in normalized endothelial resistance across time, two-way repeated measures ANOVA with* post hoc* Bonferroni's multiple comparisons tests were performed. Fold change in apoptosis and necrosis (flow cytometry and caspase-3/7 activity) following exposure of BMEC to treatments (e.g., 13-HPODE) relative to 0% FBS media control was determined by ordinary one-way ANOVA with Tukey's* post hoc* correction. Effect of several doses of N-acetylcysteine coexposure with 13-HPODE was also tested by ordinary one-way ANOVA with Tukey's* post hoc* correction. Significance set at *P* ≤ 0.05 for all tests.

## 3. Results

### 3.1. *S. uberis* Exposure Induced Inflammatory Marker Expression, but Not Oxylipid Biosynthetic Enzyme Expression, in BMEC

**Figure 1:**
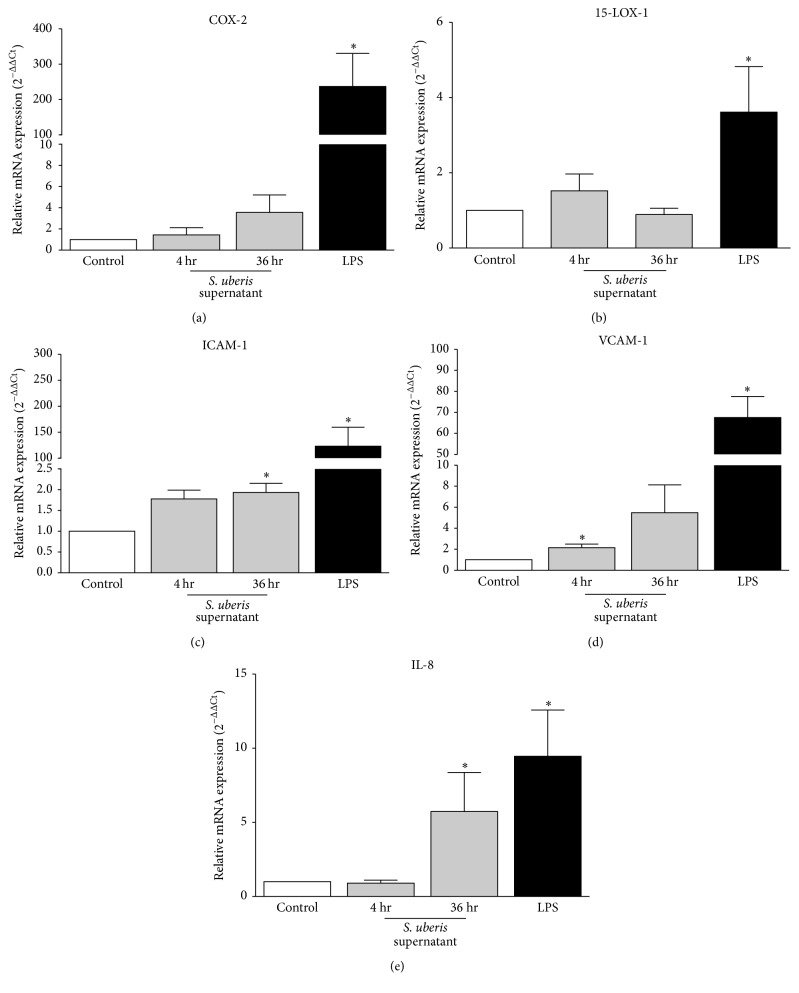
Mean changes in BMEC mRNA expression of oxylipid biosynthetic enzymes (a, b), adhesion molecules (c, d), and a chemotactic cytokine (e). Media controls are displayed as open bars. Positive control is 4 hr lipopolysaccharide (LPS) and displayed in closed bars. Light grey bars represent* S. uberis* supernatant exposure for 4 hr and 36 hr. The mRNA expression is expressed as 2^−ΔΔCt^  ± SE. Asterisks (*∗*) denote differences between media control and treatments including* S. uberis* and positive control, LPS, as tested by Student's* t*-tests. Significance declared for differences in ΔCt at *P* ≤ 0.05 (*n* = 3 for all genes).

Oxylipid biosynthetic enzyme mRNA expression (COX-2 and 15-LOX-1) was not significantly increased after 4 hr or 36 hr exposure to* S. uberis* supernatant (Figures 1(a) and 1(b)). In contrast, the mRNA expression of ICAM-1 and IL-8 was significantly increased after 4 hr exposure to* S. uberis* supernatant and VCAM-1 mRNA expression was significantly increased after 36 hr exposure (Figures 1(c), 1(d), and 1(e)). Exposure to LPS (positive control) demonstrated a significant increase in all genes tested after 4 hr (Figure 1).

### 3.2. BMEC 13-HODE and 13-oxoODE Biosynthesis Were Not Changed following* S. uberis* Exposure

**Figure 2:**
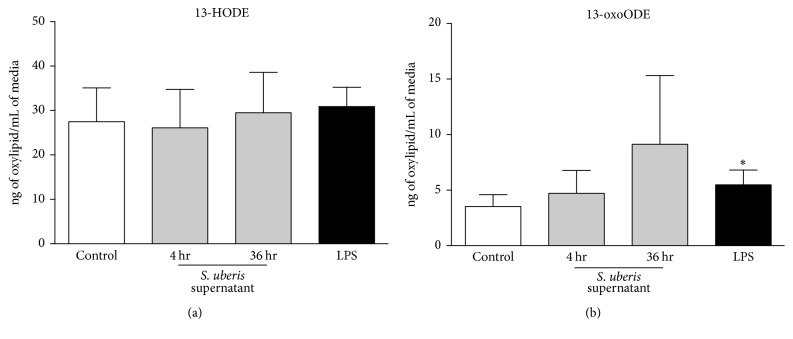
Endothelial oxylipid biosynthesis following 4 hr LPS (black bars) or* S. uberis* supernatant exposure for 4 hr and 36 hr (light grey bars) is displayed. Media controls are displayed as open bars. Mean oxylipid biosynthesis is expressed as ng of oxylipid/mL of media. Asterisks (*∗*) denote differences between media control and positive control (LPS) as tested by Student's* t*-tests. Significance for differences declared at *P* ≤ 0.05 (*n* = 3).

Exposure to* S. uberis* did not induce any significant changes in 13-HODE and 13-oxoODE biosynthesis after 4 and 36 hr (Figures 2(a) and 2(b)). Increased 13-oxoODE, but not 13-HODE, was significant after 4 hr LPS exposure (Figure 2(b)).

### 3.3. *S. uberis* Exposure Induced Expression of Inflammatory Markers, including Oxylipid Biosynthetic Enzyme Expression, in Bovine Monocytes

**Figure 3:**
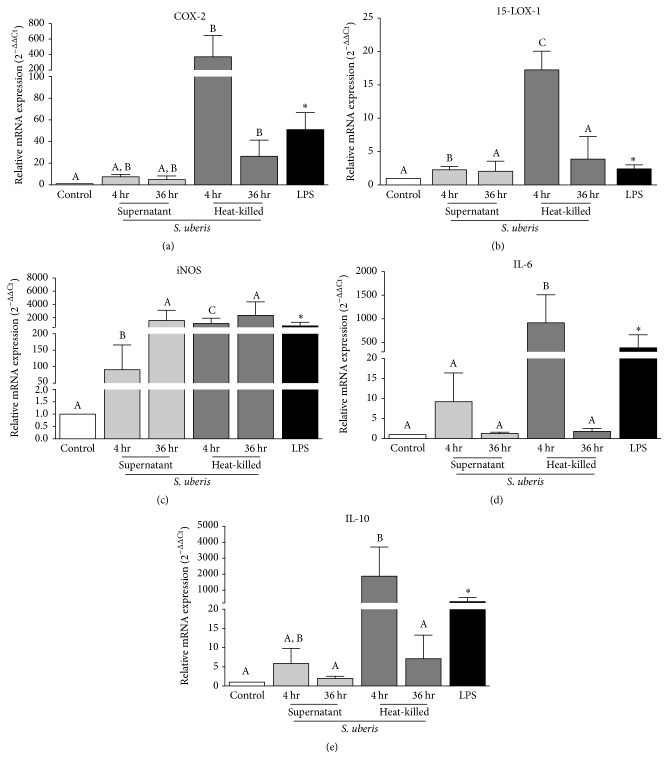
Mean changes in mRNA expression of bovine monocyte oxylipid biosynthetic enzymes (a, b), a marker of monocyte activation (c), and inflammatory cytokines (d, e). Media controls are displayed as open bars. Positive control is 4 hr LPS and displayed in closed bars. Light grey bars represent* S. uberis* supernatant exposure for 4 hr and 36 hr. Dark grey bars represent heat-killed* S. uberis* exposure for 4 hr and 36 hr. The mRNA expression is expressed as 2^−ΔΔCt^  ± SE. Asterisks (*∗*) denote differences between media control and positive control (LPS) as tested by Student's* t*-tests. Letters that differ between control time points, 4 hr and 36 hr separately, denote significant differences between control and among treatments as measured by an ordinary one-way ANOVA with Tukey's* post hoc* correction. For example, 4 hr exposure to heat-killed* S. uberis* upregulated COX-2 mRNA expression compared to control but 4 hr exposure to* S. uberis* supernatant did not (a). Additionally, COX-2 mRNA expression after heat-killed* S. uberis* exposure is not different from COX-2 mRNA expression after* S. uberis* supernatant exposure (a). A similar relationship can be described for 36 hr exposure of bovine monocytes to heat-killed and* S. uberis* supernatant. Significance declared for all tests using ΔCt at* P* ≤ 0.05 (*n* = 4 for 15-LOX-1, IL-6, and IL-10; *n* = 3 for COX-2 and iNOS).

The mRNA expression of COX-2 was significantly upregulated relative to control BMEC by the following treatments and time points: 4 hr* S. uberis* supernatant, 4 hr heat-killed* S. uberis*, and 36 hr heat-killed* S. uberis* (Figure 3(a)). The mRNA expression of 15-LOX-1 was significantly upregulated by the following treatments and time points relative to control BMEC: 4 hr* S. uberis* supernatant and 36 hr heat-killed* S. uberis* (Figure 3(b)). The mRNA expression of iNOS was significantly upregulated relative to control BMEC after 4 hr and 36 hr exposure to both* S. uberis* supernatant and heat-killed* S. uberis* (Figure 3(c)). The mRNA expression of IL-6 was significantly upregulated by the following treatments and time points relative to control BMEC: 4 hr* S. uberis* supernatant and 36 hr heat-killed* S. uberis* (Figure 3(d)). The mRNA expression of IL-10 was significantly upregulated relative to control BMEC after 4 hr exposure to heat-killed* S. uberis* (Figure 3(e)). Exposure to LPS demonstrated a significant increase in all genes tested after 4 hr (Figure 3).

### 3.4. 13-HODE, but Not 13-oxoODE, Biosynthesis by Bovine Monocytes Was Upregulated following Heat-Killed* S. uberis* Exposure

**Figure 4:**
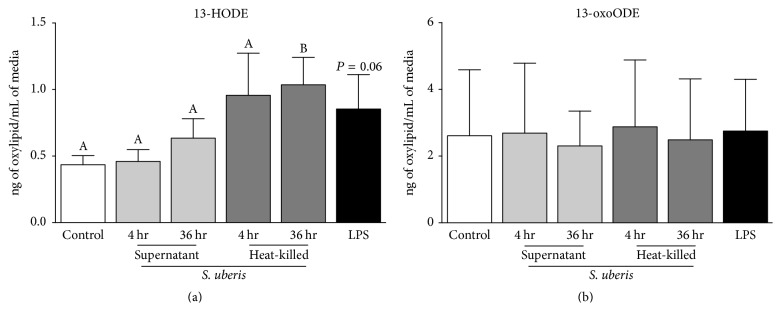
Monocyte oxylipid biosynthesis following 4 hr LPS (black bars) or* S. uberis* exposure. Media controls are displayed as open bars. Light grey bars represent* S. uberis* supernatant exposure for 4 hr and 36 hr. Dark grey bars represent heat-killed* S. uberis* exposure for 4 hr and 36 hr. Mean oxylipid biosynthesis is expressed as ng of oxylipid/mL of media. Letters that differ between control time points, 4 hr and 36 hr separately, denote significant differences between control and among treatments as measured by an ordinary one-way ANOVA with Tukey's* post hoc* correction. For example, 4 hr exposure to* S. uberis* supernatant or heat-killed* S. uberis* did not modify 13-HODE biosynthesis, whereas 36 hr exposure to heat-killed* S. uberis* did increase 13-HODE biosynthesis. No letters are displayed in (b) as no differences were detected in any time points. Significance for differences declared at *P* ≤ 0.05 (*n* = 4).

Increased 13-HODE, but not 13-oxoODE, was significant after 36 hr heat-killed* S. uberis* exposure (Figure 4(a)). Exposure to* S. uberis* supernatant for 4 and 36 hr failed to induce significant changes in 13-HODE and 13-oxoODE biosynthesis (Figures 4(a) and 4(b)). Similarly, exposure of bovine monocytes to LPS for 4 hr did not induce significant changes in 13-HODE and 13-oxoODE biosynthesis (Figures 4(a) and 4(b)).

### 3.5. Mammary Endothelial Barrier Integrity Is Decreased during 13-HPODE Treatment

**Figure 5:**
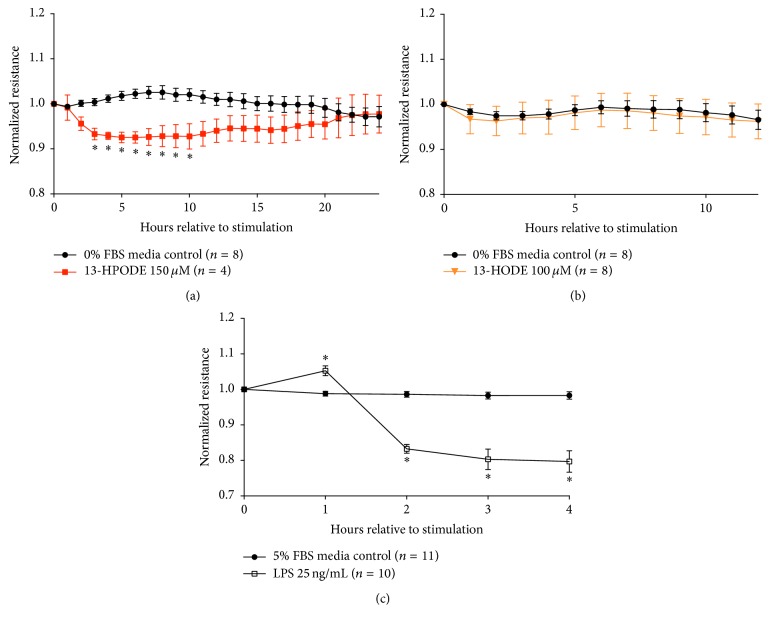
Mean normalized endothelial resistance across time during media control (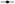) and 150 *μ*M 13-HPODE (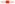) exposure is displayed in (a). Mean normalized resistance for 100 *μ*M 13-HODE (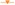) exposure up to 6 hr is displayed in (b). Mean normalized resistance across time during media control (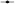) and 25 ng/mL LPS (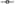) is displayed in (c). Resistance of BMEC monolayer across time for each treatment group (control and 13-HPODE, or control and 13-HODE, or control and LPS) was normalized relative to time 0. Significance of differences in resistance across time between treatment groups was tested by a two-way repeated measures ANOVA and adjusted by Bonferroni's multiple comparisons test. An asterisk (*∗*) represents significance declared at *P* ≤ 0.05.

Cultured endothelial barrier integrity was significantly decreased by 150 *μ*M 13-HPODE from 2 hr after exposure until 12 hr after exposure compared to media control at the respective time points (Figure 5(a)). 6 hr time point and 24 hr time point were used for subsequent determination of apoptosis and necrosis following 13-HPODE treatment. Barrier integrity was unchanged during exposure to 100 *μ*M 13-HODE (Figure 5(b)). Treatment of endothelial monolayers with 25 ng/mL LPS was the positive control for all ECIS experiments. Treatment with LPS consistently reduced barrier integrity within 2 hr posttreatment application (Figure 5(c)).

### 3.6. Apoptosis and Necrosis of BMEC Were Increased during 13-HPODE Treatment

**Figure 6:**
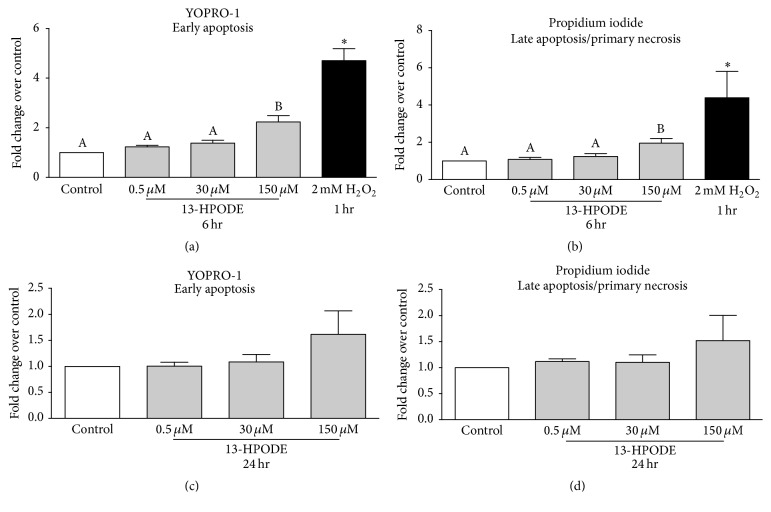
YOPRO®-1 and propidium iodide of BMEC staining displayed as fold change over media control after 6 hr ((a) and (b)) and 24 hr ((c) and (d)) 13-HPODE exposure. Media controls are displayed in open bars and 13-HPODE treatments are in light grey bars. Positive control (1 hr 2 mM H_2_O_2_ exposure) YOPRO-1 and propidium iodide staining are displayed in (a) and (b) and are the black bars. Different letters demonstrate a significant difference among media control and 13-HPODE treatments determined by one-way ANOVA with Tukey's* post hoc* correction. An asterisk (*∗*) represents a significant difference between medial control and H_2_O_2_ as determined by Student's* t*-tests. Significance declared at *P* ≤ 0.05.

**Figure 7:**
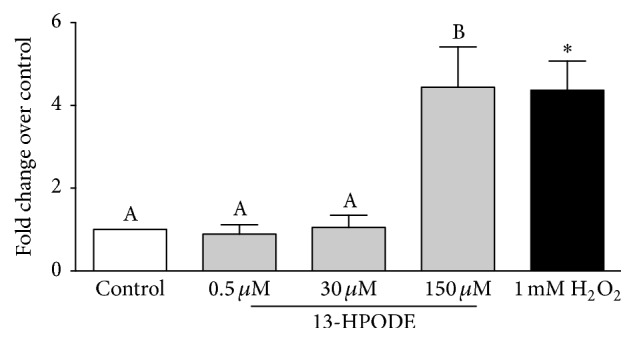
Mean change in caspase-3/7 activity of BMEC displayed as fold change over media control (open bar) after 6 hr 13-HPODE (light grey bars) or 1 mM H_2_O_2_ exposure for 6 hr (black bar, positive control). Different letters demonstrate a significant difference among media control and 13-HPODE treatments determined by one-way ANOVA with Tukey's* post hoc* correction. An asterisk (*∗*) represents a significant difference between medial control and H_2_O_2_ as determined by Student's* t*-tests. Significance declared at *P* ≤ 0.05.

Exposure of BMEC to 150 *μ*M 13-HPODE for 6 hr significantly increased YOPRO-1 staining over control indicating an increase in early apoptosis, whereas 0.5 *μ*M and 30 *μ*M 13-HPODE did not (Figure 6(a)). Similarly, exposure of BMEC to 150 *μ*M 13-HPODE for 6 hr significantly increased propidium iodide staining over control indicating an increase in late apoptosis/primary necrosis, but 0.5 *μ*M and 30 *μ*M 13-HPODE did not (Figure 6(b)). There were no significant differences in apoptosis or necrosis after 24 hr 13-HPODE exposure (Figures 6(c) and 6(d)). Exposure to 2 mM H_2_O_2_ for 1 hr (positive control) demonstrated a significant increase in YOPRO-1 and propidium iodide staining (Figures 6(a) and 6(b)). Exposure of BMEC to 150 *μ*M 13-HPODE for 6 hr significantly increased caspase-3/7 activity but 0.5 *μ*M and 30 *μ*M did not (Figure 7). Exposure to 1 mM H_2_O_2_ for 6 hr (positive control) demonstrated a significant increase in caspase-3/7 activity (Figure 7).

### 3.7. N-Acetylcysteine Ameliorates 13-HPODE-Induced Apoptosis and Necrosis of BMEC

**Figure 8:**
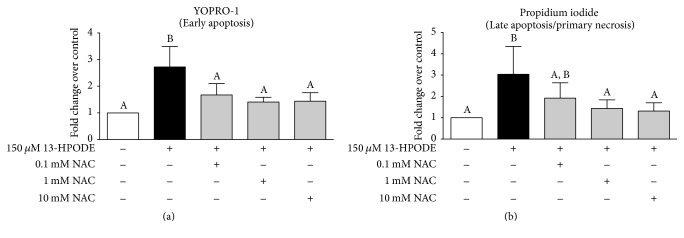
YOPRO-1 and propidium iodide of BMEC staining displayed as fold change over media control (open bar) after 6 hr 150 *μ*M 13-HPODE (light grey bars) or 6 hr coexposure with 150 *μ*M 13-HPODE or N-acetylcysteine (NAC, light grey bars). Different letters demonstrate a significant difference among media control and treatments as determined by a one-way ANOVA with Tukey's* post hoc* correction. Significance declared at *P* ≤ 0.05.

Coexposure of BMEC to 150 *μ*M 13-HPODE and 3 doses of N-acetylcysteine (0.1 mM, 1 mM, and 10 mM) prevented 13-HPODE-induced early apoptosis as demonstrated by decreased YOPRO-1 staining over control (Figure 8(a)). Coexposure of BMEC to 150 *μ*M 13-HPODE and 2 doses of N-acetylcysteine (1 mM and 10 mM) prevented 13-HPODE-induced late apoptosis/early necrosis as demonstrated by decreased propidium iodide staining over control (Figure 8(b)).

### 3.8. N-Acetylcysteine Rescues 13-HPODE-Induced Impairment of Endothelial Integrity

**Figure 9:**
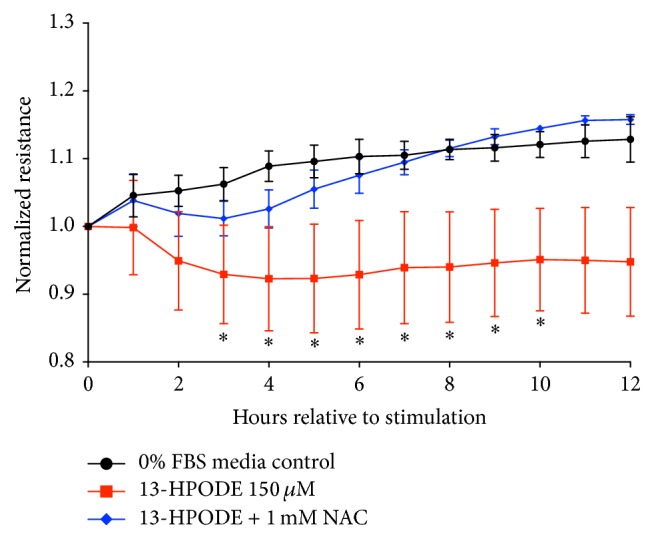
Mean normalized endothelial resistance across time during media control (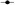), 150 *μ*M 13-HPODE (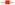), and 150 *μ*M 13-HPODE + 1 mM N-acetylcysteine (NAC) (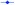) exposure is displayed in Figure 9. Resistance of BMEC monolayer across time for each treatment group (control, 13-HPODE, and 13-HPODE + NAC) is normalized relative to time 0. Significance of differences in resistance across time between treatment groups was tested by a two-way repeated measures ANOVA and adjusted by Bonferroni's multiple comparisons test. An asterisk (*∗*) represents significance declared at *P* ≤ 0.05. There were no significant differences detected between media control and 13-HPODE + 1 mM NAC.

Coexposure of BMEC monolayer to 150 *μ*M 13-HPODE and 1 mM N-acetylcysteine prevented a significant decrease in endothelial barrier integrity compared to exposure of 150 *μ*M 13-HPODE alone (Figure 9).

## 4. Discussion

Endothelial cells play an active role during an inflammation response to bacteria and bacterial products. Consistent with increased adhesion molecule expression in* S. uberis*-infected mammary tissue, our data demonstrated increased ICAM-1 and VCAM-1 expression in BMEC following exposure to* S. uberis* supernatant [17]. The observed increase in adhesion molecule expression confirms that BMEC are capable of responding to* S. uberis* exposure. One way in which activated endothelial cells may mediate the inflammatory response is through the enzymatic production of oxylipids through the COX and LOX enzymatic pathways [5, 30, 31]. In contrast to the observed increases in adhesion molecule transcripts, however, expression of COX-2 and 15-LOX-1 mRNA in BMEC was not significantly changed by* S. uberis* exposure [17]. Previous murine studies suggested that COX-2 expression was dependent on recognition of pathogen associated molecular patterns by host pathogen receptors, such as Toll-like receptors (TLR) [32]. In the case of* S. uberis*, a recent study demonstrated an inability of heat-killed and live* S. uberis* to induce TLR-2 signaling in mammary epithelial cells [33]. Though our study did not evaluate TLR activity, failure to activate the BMEC TLR-2 pathway could result in a failure to induce significant expression of oxylipid biosynthetic enzymes, especially COX-2 and 15-LOX-1. Consistent with no change in 15-LOX-1 mRNA expression after* S. uberis* supernatant exposure, BMEC did not significantly increase LA-derived oxygenation products from the 15-LOX-1 pathway. Additionally, the oxylipid profiles induced by BMEC during* S. uberis* exposure did not mimic previously described* S. uberis*-infected mammary tissue profiles [17]. Most importantly, biosynthesis of 13-HODE by BMEC was not increased by* S. uberis* supernatant exposure suggesting that BMEC may not be an important source of oxylipids derived from LA metabolism through the 15-LOX-1 pathway during* S. uberis* mastitis.

Demonstrating that endothelial cells may not be an important source of 13-HODE during* S. uberis* mastitis required evaluation of other potential cellular sources of oxylipid biosynthesis during an inflammatory response. In murine studies, the macrophages demonstrated the highest expression of 12/15-LOX and represented a significant source of 15-LOX-derived oxylipids [28, 34]. Resident macrophages also are instrumental in alerting the surrounding mammary cells that a perceived insult is present [35]. Therefore, we developed a primary bovine monocyte* S. uberis* challenge model and showed that heat-killed* S. uberis* increased both 15-LOX-1 expression and 13-HODE biosynthesis. Current findings were in contrast to* in vivo S. uberis* intramammary challenge, which did not demonstrate a significant increase in 15-LOX-1 mRNA expression in* S. uberis*-infected mammary tissue [17]. Collection of* S. uberis*-infected tissue may have occurred after peak 15-LOX-1 expression because increased 13-HODE biosynthesis in* S. uberis*-infected mammary tissue supported increased 15-LOX-1 activity [17]. Additionally, the relative contribution of macrophages expressing 15-LOX-1 compared to mammary epithelial cells and infiltrating neutrophils in infected tissue was not known [3]. Heat-killed* S. uberis* exposure of bovine monocytes induced a significant increase in 13-HODE biosynthesis and may suggest that optimal monocyte 15-LOX-1 activation requires recognition of bacteria by host pathogen recognition receptors [33]. For example,* S. uberis* supernatant in the current study failed to induce a significant increase in 13-HODE biosynthesis in bovine monocytes as well as BMEC. Nonetheless, our data suggested that bovine monocytes represented a more robust source of both 13-HPODE and 13-HODE during heat-killed* S. uberis* exposure when compared to mammary endothelial cells. A better understanding of cellular sources of potent lipid mediators within an inflammatory foci could lead to more targeted therapies for the control of certain inflammatory based diseases such as mastitis.

Although LA-derived oxylipids from 15-LOX-1 metabolism were shown to be increased significantly during* S. uberis* mastitis [17], the relative contribution of individual oxylipids to vascular dysfunction has not been identified previously. Our results demonstrated for the first time the capability of 13-HPODE, but not the reduced hydroxyl (13-HODE), to modify endothelial monolayer integrity. These findings are not consistent with a previous study that demonstrated that the arachidonic acid-derived hydroxyl (15-hydroxyicosatetraenoic acid, 15-HETE) was capable of reducing bovine microvascular retinal endothelial barrier integrity [36]. In addition to the different substrates from which 15-HETE and 13-HODE are derived, the difference between the effects of arachidonic acid-derived hydroxyls and 13-HODE on barrier integrity could be due to the source of endothelial cells. In this study, BMEC were obtained from the macrovasculature, whereas retinal endothelial cells were isolated from the microvasculature [37]. Thus, the possibility exists that both the fatty acid source of oxylipids and the location of endothelial cells will determine the relative impact of lipid metabolism through the 15-LOX-1 pathway on vascular barrier integrity.

In contrast to 13-HODE, however, we showed that 13-HPODE reduced mammary endothelial barrier functions, but the reduction was not sustained throughout the duration of the treatment period. These observations suggest an ability of the endothelial cells to overcome the adverse effects of 13-HPODE and that any loss in vascular barrier function may be reversible. During an inflammatory response to bacterial infection, however, there could be repeated exposure to newly synthesized 13-HPODE that may not allow the endothelial barrier to recover. Though we anticipated that 13-HPODE would decrease barrier integrity, confirmation of 13-HODE being unable to modify barrier integrity in our model was essential. Reduction of 13-HPODE generates 13-HODE, and treatment with 13-HPODE would most likely contribute to increased exposure to 13-HODE as well. Other studies have showed differential effects of 13-HPODE and 13-HODE on endothelial activation, but the present study was the first to demonstrate different effects on endothelial monolayer integrity [13]. In support of compromised barrier integrity, additional functional studies would be useful to evaluate how 13-HPODE may contribute to enhanced vascular permeability to macromolecules or uncontrolled leukocyte transmigration.

Apoptosis and necrosis of endothelial cells disrupt the continuous, single-cell layer necessary for orchestrating an effective and self-limiting inflammatory response. The current study demonstrated induction of apoptosis and necrosis of BMEC in conjunction with decreased mammary endothelial barrier integrity. Our findings were consistent with previous work that demonstrated 13-HPODE-induced apoptosis in bovine aortic endothelial cells [19–21]. Though not evaluated in the current study, previous data suggested that 13-HPODE activated the intrinsic pathway for apoptosis as a result of mitochondrial dysfunction [19, 20]. For example, exposure of bovine aortic endothelial cells to 13-HPODE increased the activity of intrinsic pathway caspases 3 and 9 and was associated with a loss in mitochondrial function [20]. The primary proposed mechanism by which 13-HPODE can induce apoptosis is by lipid peroxidation of cell membranes [38]. Phospholipids in cell membranes contain an abundance of esterified PUFA and are extremely susceptible to lipid hydroperoxide attack. Propagation of lipid peroxidation occurs by decomposition of lipid hydroperoxides by transition of metals to generate lipid alkoxyls (LO^*∙*^) and lipid peroxyl radicals (LOO^*∙*^) [39]. The lipid peroxyl radicals can act as prooxidants and attack membrane-esterified PUFA to generate additional LO^*∙*^, LOO^*∙*^, and LOOH, such as 13-HPODE. Peroxidation of lipid membranes initiated by extracellular 13-HPODE may contribute to an irreversible increase in mitochondrial permeability and permanent loss of function [39]. Thus preventing accumulation of lipid peroxides and other prooxidants may be protective or beneficial in limiting the effects of 13-HPODE [40]. Overall, the data from the current study demonstrated that shifting oxidant status in the endothelial microenvironment during 13-HPODE treatment by promoting reduction of prooxidants with an antioxidant may limit initiation and propagation of lipid peroxidation, thus preventing apoptosis and impaired barrier integrity.

## 5. Conclusions 

The tightly regulated, self-limiting inflammatory response is dependent on optimal endothelial function and maintenance of the endothelial barrier. Some oxylipids may reduce the ability of endothelial cells to orchestrate an optimal inflammatory response. Thus, our study defined a potential source and role for LA-derived 15-LOX-1 metabolites. Our data showed that bovine monocytes, but not BMEC, may be an important source of 15-LOX-1 oxygenation products of LA during* S. uberis* exposure. Furthermore, exposure of BMEC to 13-HPODE, but not 13-HODE, contributed to impaired mammary endothelial barrier integrity and apoptosis of BMEC. We also showed that oxidant status during 13-HPODE treatment may contribute to cell death and barrier integrity. Elucidating the mechanisms by which oxidant status mediates vascular function may be critical to developing targeted therapies for bovine mastitis.

## Figures and Tables

**Table 1:** Bovine primers for qRT-PCR.

Gene^a^	Reference sequence	TaqMan assay ID^b^

*TBP*	NM_001075742.1	Bt03241946_m1
*PGK1*	NM_001034299.1	Bt03225857_m1
*ACTB*	NM_173979.3	Bt03279174_g1
*ICAM-1*	NM_174348.2	Bt03213911_m1
*VCAM-1*	NM_174484.1	Bt03279189_m1
*IL-6*	NM_173923.2	Bt03211905_m1
*IL-8*	NM_173925.2	Bt03211906_m1
*IL-10*	NM_174088.1	Bt03212727_m1
*iNOS*	M_001076799.1	Bt03249581_m1
*15-LOX-1*	NM_174501.2	Bt03214775_m1
*COX-2*	NM_174445.2	Bt03214492_m1

^a^TBP: TATA-box binding protein; PGK1: phosphoglycerate kinase; ACTB: *β*-actin; ICAM-1: intercellular adhesion molecule-1; VCAM-1: vascular adhesion molecule-1; IL: interleukin, iNOS: inducible nitric oxide synthase; 15-LOX-1: 15-lipoxygenase-1; COX-2: cyclooxygenase-2.

^b^Applied Biosystems Foster City, CA.
